# Racial and Ethnic Disparities in Viral Acute Respiratory Infections in the United States: Protocol of a Systematic Review

**DOI:** 10.21203/rs.3.rs-121890/v1

**Published:** 2020-12-08

**Authors:** Neia Prata Menezes, Jowanna Malone, Carrie Lyons, Kechna Cadet, Lorraine Dean, Gregorio Millett, Stefan Baral

**Affiliations:** Johns Hopkins University Bloomberg School of Public Health; Johns Hopkins University Bloomberg School of Public Health; Johns Hopkins University Bloomberg School of Public Health; Johns Hopkins University Bloomberg School of Public Health; Johns Hopkins University Bloomberg School of Public Health; amfAR: Foundation for AIDS Research; Johns Hopkins University Bloomberg School of Public Health

**Keywords:** acute respiratory infection, racial disparities, health disparities, influenza like illness, pandemic preparedness, pandemic response, structural racism

## Abstract

**Background:**

The COVID-19 pandemic caused by SARS-CoV-2 has highlighted consistent inequities in the risk of infection, severity of disease, or mortality across racial and ethnic minority populations in the United States and beyond. Although novel, SARS-CoV-2 shares commonalities in transmission dynamics with other viral respiratory pathogens where similar disparities in morbidity and mortality have been documented. However, to date, there has not been a systematic review of disparities in viral respiratory pathogens. In response, this review aims to synthesize data on racial and ethnic disparities in morbidity and mortality due to viral acute respiratory infections (ARI) other than SARS-CoV-2. In particular, this review will focus on understanding structural health and social factors outside of race and ethnicity driving these disparities in the United States.

**Methods:**

We will conduct a systematic review of studies published between January 1, 2002 and September 30, 2020 that capture data on racial and ethnic disparities associated with increased incidence, disease severity, risk of hospitalization and/or death in viral ARI in the United States. Data characterizing individual-, community-, and structural-level factors associated with these disparities will be abstracted to better understand the underlying structural inequities contributing to racial disparities in ARI. The Preferred Reporting Items for Systematic Reviews and Meta-Analyses (PRISMA) guidelines will be used with reviewers employing COVIDENCE to conduct two independent rounds of title/abstract and full text reviews for all articles. A built-in tool in COVIDENCE will be used for data abstraction.

**Discussion:**

Findings from this systematic review will shed light on patterns of racial and ethnic disparities in viral ARI in the United States. Leveraging these data can support predictive studies of the differential impacts of COVID-19 across the United States as well as adaptive intervention strategies mitigating structural inequities, including structural racism, driving both incidence and disparities in marginalized communities. Moreover, data emerging from this review may reignite pandemic preparedness focused on vulnerable communities given structural inequities, facilitating improved future pandemic responses to novel or endemic viral respiratory pathogens in the United States.

**Systematic review registration:**

PROSPERO CRD42020219771

## Background

Emerging evidence has highlighted the stark racial and ethnic disparities in the risk of infection and death caused by the novel severe acute respiratory syndrome coronavirus 2 (SARS-CoV-2), the virus responsible for COVID-19. In the United States, African American, Latinx, and indigenous populations comprise a disproportionate number of COVID-19-related deaths (1–4). Notably, these disparities have mirrored trends observed in prior epidemics of other viral acute respiratory infections (ARI). During the 1918 influenza pandemic, Black Americans were more likely to die compared to their White counterparts (5). Most recently, during the 2009 H1N1 influenza pandemic, minority groups were more likely to be hospitalized and to die compared to non-Hispanic white populations (6, 7). The purpose of this systematic review is to investigate racial and ethnic disparities in morbidity and mortality of prior outbreaks, epidemics, and pandemics of ARI to provide context in understanding the disparate impact of the current COVID-19 epidemic across the United States.

Despite the growing interest to explore literature concerning racial disparities and social determinants of ARI, there are no systematic reviews comprehensively characterizing research focused on racial and ethnic disparities from former infectious disease outbreaks of viral respiratory pathogens (8). Aside from recent literature concerning SARS-CoV-2, there is a lack of research documenting racial disparities in prior outbreaks or pandemics. However, there is existing evidence of racial disparities involving ARI. In the United States, research has shown that Black children have higher rates of hospitalizations from ARI compared to white children, although they do not differ in severity of disease once hospitalized (9). There has also been evidence that acute infections can contribute to racial disparities in stroke-related deaths; however, this included non-respiratory tract infections in addition to ARI (10).

Considering the ever-growing focus on the contribution of racial inequities to multiple facets of health, and the present burden of COVID-19-related deaths on African-American, Latinx, and indigenous communities in the current pandemic, it is imperative to understand factors, including structural racism, that have contributed to these disparities in earlier outbreaks. Such knowledge would inform development of a pandemic preparedness response that ensures protection of the most vulnerable.

Framing pandemic preparedness through a social justice lens is not a novel concept and has been previously emphasized as integral to mounting an effective response strategy (11–13). Nevertheless, underscoring the protection of groups at highest risk of disease burden based on socioeconomic factors has yet to be incorporated into existing national strategies (14). By failing to protect the most vulnerable, we not only miss the opportunity to prevent and mitigate disease burden among those at highest risk and in the population as a whole, but also inadvertently perpetuate the systemic inequities that contribute to ongoing health disparities in minority communities. A deepened understanding of the racial disparities in ARI attributable to structural and systemic inequities will not only prevent further disparities from occurring in the event of future outbreaks or pandemics but can also alleviate present disparities in endemic ARI.

## Objectives

This systematic review will assess data published between January 1, 2002 and September 30, 2020 that characterizes the racial and ethnic disparities associated with increased incidence, disease severity, risk of hospitalization, or death in viral acute respiratory infections, other than SARS-CoV-2, in the United States. We will additionally summarize data in this same time period that describes the individual-, community-, and structural-level factors correlated with race/ethnicity (e.g. socio-economic status, geography/neighborhood, healthcare accessibility) that are associated with increased morbidity and mortality in acute respiratory infections.

## Objective

- To complete a systematic review of available data capturing racial and ethnic disparities in the incidence, morbidity, and mortality of viral acute respiratory infections from 2002 to 2020 in the United States.
- To determine which racial/ethnic populations are disproportionately affected and evaluate underlying drivers of these disparities including structural racism

## Primary outcomes

1. ARI incidence
2. Disease severity/complication due to ARI
3. Hospitalization due to ARI
4. Death attributed to ARI

Secondary outcomes of this review will assess uptake of preventive interventions shown to be effective for mitigating the spread of viral respiratory pathogens. These include vaccine coverage and uptake, handwashing, social distancing, and wearing masks (15).

## Study Methodology

This protocol is registered under the PROSPERO database (CRD42020219771) and designed in accordance with standardized guidelines specified in the Preferred Reporting Items for Systematic Reviews and Meta-analyses (PRISMA) (see Additional file 1) (16). Findings will be reported using the PRISMA checklist and any amendments to the protocol will be documented in the final review.

Inclusion & exclusion criteria

To be included in the review, an article must meet the following criteria:

1. Studies will capture data on persons of any age who are at risk for, or infected with, a respiratory viral pathogen (influenza virus, respiratory syncytial virus (RSV), middle east respiratory syndrome (MERS), severe acute respiratory syndrome coronavirus 1 (SARS), parainfluenza virus, measles virus, rubella virus, rhinovirus, and/or adenovirus).
2. Studies will measure the following as an exposure: Non-white race/ethnicity, such as Black/African American, Asian, American Indian/Alaska Native, Native Hawaiian/other Pacific Islander, non-white Hispanic or Latinx, as defined by the United States Census Bureau (17).
3. Studies will measure one or more of the primary or secondary outcomes listed above.
4. Studies were publications in peer-reviewed journals or an abstract at a conference with peer-reviewed blinded abstract selection process.
5. At a minimum, studies captured and reported information on racial/ethnic disparities in ARI and listed details regarding data sampling techniques.
6. Articles published since January 1, 2002 with data collection started no later than January 1, 2000.
7. All studies were conducted in English and based in the United States

Articles will be excluded based on the following criteria:

1. Study only addresses disparities with the novel SARS-CoV-2 that causes COVID-19
2. Study was conducted outside of the United States
3. Study conducted among non-human, animal subjects
4. Sample size is less than 100 persons
5. Study does not mention race/ethnicity in the abstract
6. Study is based on modelling data

Study designs

We will include primary studies of any design that describe racial/ethnic disparities associated with respiratory infections due to influenza virus, RSV, MERS, SARS, parainfluenza virus, measles virus, rubella virus, rhinovirus, and/or adenovirus. Studies will be included when non-white race/ethnicity is compared to the following: no comparison; white race/ethnicity; other non-white race/ethnicity

Information sources and search strategies

In partnership with an information specialist at Johns Hopkins University, we will search the following electronic databases: the National Library of Medicine’s MEDLINE database using the PubMED interface, EBSCO Host- CINAHL Plus, PsycInfo, EMBASE, and Cochrane Library. Search criteria and terms were created based on validated peer-reviewed systematic reviews and articles regarding racial disparities and acute respiratory infections (8). We will search the gray literature by hand. Reference lists of identified articles and reports will be reviewed for additional articles.

We piloted multiple search strategies to optimize an approach that is highly sensitive yet prioritizes identifying relevant articles. The search strategies are comprised of a combination of controlled MeSH terms and other search terms that cover two independent concepts: acute respiratory infections and racial/ethnic disparities (see Additional file 2). Search terms pertaining to acute respiratory infections include terms relating to viral infections due to influenza, other coronaviruses (excluding SARS-CoV-2), other viral respiratory pathogens, and general terms for acute respiratory infection and/or influenza like illness. Terms associated with racial disparities include terms relating to general health disparities or inequities, race/ethnicity, socioeconomic factors (e.g. poverty, education), and historically marginalized communities (undocumented immigrants, incarcerated persons). We opted to not include search terms specific to racial/ethnic groups to mitigate the potential for missing articles due to improper indexing. Additionally, by including terms that are not limited to racial/ethnic groups (e.g. poverty, health care access), we hope to capture information on disparities associated with structural and systemic inequities, including structural racism, that overwhelmingly affect racial and ethnic minority communities in the United States.

Study selection & quality assessment

The literature review consists of an iterative title review, abstract review, and full text review for articles that meet the criteria (18). Two reviewers conduct parallel screening of titles found in the search. If either one or both of the two reviewers selects a title to move forward to abstract review, the abstract will be reviewed independently and assessed for inclusion in the full article review. If either one or both of reviewers selects the abstract for full article review, the article will be selected for full article review. If at the full article review there is a disagreement between the first two reviewers regarding data extraction, a third reviewer will review and solve the disagreement. All articles selected for review will be assessed for risk of bias using the Newcastle-Ottawa Scale, a quality assessment tool for non-randomized studies, that assesses articles according to three components: study group selection, group comparability, and outcome ascertainment (19).

Strategy for data extraction

Data will be extracted for each full article reviewed and entered into a pre-piloted data collection form based on COVIDENCE—a commercially available web-based tool for conducting and managing systematic reviews. Reviewers will be trained to abstract the relevant data from the articles using the data abstraction tool.

The following fields will be abstracted from all included studies:

- Source reference
- Source type (e.g. journal article, abstract) and publication year
- Population (adult ≥ 18, child < 18, and any qualifying characteristics such as race/ethnicity, sex/gender, etc.)
- Geographic setting (within the United States)
- Sample size
- Nature of study (descriptive, quantitative, qualitative)
- Study design (e.g. cross-sectional, etc.)
- Individual-, community-level, or structural-level factors discussed
- Type of individual-, community-, or structural-level factor discussed (e.g. race/ethnicity, socioeconomic status, healthcare coverage, housing, citizenship status, neighborhood location, food security)
- Measures of effect for all primary or secondary outcomes of interest, including proportions, relative risks, odds ratios, or hazard ratios if time series data available
- Summary of author interpretations/conclusions

Synthesis and feasibility for meta-analysis

Data will be analyzed according to study outcomes and race/ethnicity exposures. For qualitative research, we will present descriptive summaries, and if race/ethnicity exposures are similarly measured, we will assess consistency of thematic results. For quantitative studies reporting the same outcome, among populations deemed suficiently similar, we will conduct meta-analysis using random effects meta-analytic models. We will evaluate heterogeneity within selected studies by examining forest plots and conducting Cochrane’s Q and I2 statistical tests. We will assess the quality of the body of evidence contributing to the pooled effect estimate for each outcome using criteria recommended by the GRADE Working Group: GRADE evidence certainty for individual outcomes (20–25). Funnel plots will be used to assess for risk of publication bias where 10 or more similar studies are included.

## Discussion

The aim of this review is to investigate disparities in morbidity and mortality in acute respiratory viral infections other than SARS-CoV-2. Some practical challenges are expected in the identification of existing evidence. First, the medical library indexing of articles may influence the results obtained in this review. It is common practice to report outcomes by race, however, if differential outcomes are not a primary objective of the study, the article may not be indexed in the medical library accordingly. Therefore, these studies may not be represented in this review.

Second, racial and ethnic disparities are documented to be associated with interrelated socioeconomic factors representing macrolevel inequalities such as income, neighborhood, stigma and discrimination, and barriers or access to healthcare (11). Given this, studies may focus either on upstream determinants of racial disparities, or manifestations of structural inequalities other than race. The search protocol has been developed to consider this complexity; however discerning intersecting factors is an expected challenge of this review. Lastly, under-representation of racial and ethnic minorities in clinical trials has been documented in other contexts (26). Specifically, historical ethics violations of human subjects’ research have disproportionately affected minorities in the United States, and potentially deterred study participants from minority communities. Therefore, study populations may not be representative of racial and ethnic minorities. These potential challenges may limit the comprehensiveness of this review and therefore limit the conclusions.

The findings from this systematic review will provide context and insight to understand patterns of disparity in viral acute respiratory infections in the United States. Furthermore, this review may support a robust comprehension of the manifestations of structural and social inequities affecting racial and ethnic minorities and their influence on ARI outcomes. Leveraging this information could influence development and implementation of studies on the differential impact of COVID-19 across the United States. Moreover, this information could be used to inform the development of strategies to alleviate the structural inequities driving racial and ethnic disparities prevalent in ARI. Finally, the newly elected President- and Vice President-elect recently proposed the establishment of a COVID-19 Racial and Ethnic Disparities Task Force as an integral component of their plans to address the ongoing COVID-19 pandemic (27). We hope that this review adds to the body of evidence that will drive future decision-making of this task force by reigniting a focus on pandemic preparedness and public health response to other ARI that underscores the protection of vulnerable communities, given systemic structural inequities.

## Supplementary Material

Supplement

Supplement

## Data Availability

All data will be made available upon the completion of the review process.

## Abbreviations

ARI: Acute respiratory infection
ILI: Influenza-like illness
RSV: respiratory syncytial virus
MERS: Middle East Respiratory Syndrome
SARS: Severe acute respiratory syndrome
SARS-CoV-2: Severe acute respiratory syndrome coronavirus 2
COVID-19: Coronavirus disease 2019
