# Supplementary material for: Racial and Ethnic Disparities in Viral Acute Respiratory Infections in the United States: Protocol of a Systematic Review
Source: Res Sq. 2020 Dec 8:rs.3.rs-121890. Preprint. [Version 1] doi: 10.21203/rs.3.rs-121890/v1 (PMC7743074; doi:10.21203/rs.3.rs-121890/v1)
Supplement: Supplement [file 73ab9c04d1fa4bf5e35ddd33.docx]

**Additional File 2: Draft Medline Search Terms**

**Acute respiratory infection**

*Influenza-related*

"Influenza, Human"[Mesh] OR influenza[Text Word] OR influenza in human[Text Word] OR "Influenza A Virus, H1N1 Subtype"[Mesh] OR h1n1 virus[Text Word] OR "pandemics"[MeSH Terms] OR pandemic[Text Word] OR

*Other coronaviruses*

"Middle East Respiratory Syndrome Coronavirus"[Mesh] OR MERS[Text Word] OR middle east respiratory syndrome[Text Word] OR "Severe Acute Respiratory Syndrome"[Mesh] OR "SARS Virus"[Mesh] OR severe acute respiratory syndrome[Text Word] OR SARS[Text Word] OR sars coronavirus[Text Word] OR "coronavirus infections"[MeSH Terms] OR

*Other ARI*

"respiratory syncytial viruses"[MeSH Terms] OR respiratory syncytial virus[Text Word] OR "paramyxoviridae infections"[MeSH Terms] OR parainfluenza[Text Word] OR "measles"[MeSH Terms] OR measles[Text Word] OR "rubella"[MeSH Terms] OR rubella[Text Word] OR "rhinovirus"[MeSH Terms] OR rhinovirus[Text Word] OR common cold[Text Word] OR "adenoviridae"[MeSH Terms] OR "adenoviridae infections"[MeSH Terms] OR adenovirus[Text Word] OR

*General ARI/ILI*

acute respiratory illness[Text Word] OR influenza like illness[Text Word]

**[AND]**

**Disparities**

*General disparities/inequities*

"health status disparities"[MeSH Terms] OR "healthcare disparities"[MeSH Terms] OR disparit*[Text Word] OR inequalit*[Text Word] OR

*Race/ethnicity*

"ethnic groups"[MeSH Terms] OR ethnic*[Text Word] OR race[Text Word] OR racial[Text Word] OR

*Socioeconomic factors*

"socioeconomic factors"[MeSH Terms] OR socioeconomic factor[Text Word] OR "Poverty"[Mesh] OR poverty[Text Word] OR "Educational Status"[Mesh] OR

*Vulnerable populations*

"Vulnerable Populations"[Mesh] OR high-risk population[Text Word] OR hard-to-reach populations[Text Word] OR "homeless persons"[MeSH Terms] OR homeless[Text Word] OR "housing"[MeSH Terms] OR housing[Text Word] OR "residence characteristics"[MeSH Terms] OR neighborhood[Text Word] OR "Undocumented Immigrants"[Mesh] OR "prisons"[MeSH Terms] OR prison[Text Word] OR correctional facility[Text word] OR structural vulnerability[Text Word] OR mass incarceration[Text Word]
